# The Bacterial Community Characteristics of *Hippophae rhamnoides* Subsp. *sinensis* Rousi in Different Distribution Areas of the Qinghai–Tibet Plateau and Their Responses to Habitat Factors

**DOI:** 10.3390/biology14091304

**Published:** 2025-09-21

**Authors:** Pei Gao, Guisheng Ye, Yuhua Ma

**Affiliations:** 1 Agriculture and Animal Husbandry College, Qinghai University, Xining 810016, Chinaqhxjygs@163.com (G.Y.); 2 Academy of Animal Husbandry and Veterinary Sciences, Qinghai University, Xining 810016, China

**Keywords:** *Hippophae rhamnoides* subsp. *sinensis* Rousi, rhizosphere soil, bacterial community, soil physicochemical properties, climatic characteristics, altitude

## Abstract

Investigating the compositional characteristics of rhizosphere soil bacterial communities of Chinese seabuckthorn across different distribution areas and their relationship with habitat factors holds significant value for the development and utilization of characteristic medicinal plant resources in the Qinghai–Tibet Plateau. This study targeted rhizosphere soil from 12 distribution areas of Chinese seabuckthorn on the Qinghai–Tibet Plateau. By integrating measurements of soil and climatic parameters with high-throughput sequencing and redundancy analysis (RDA), the research systematically elucidated the characteristics of rhizosphere bacterial communities and their environmental driving mechanisms. The results revealed that rhizosphere bacterial communities of Chinese seabuckthorn across all 12 distribution areas were dominated by Proteobacteria, Acidobacteriota, and Actinobacteriota. Among them, the R2 habitat exhibited the highest amplicon sequence variant counts (3496), while R1, R8, R9, and R12 habitats showed significantly lower counts. α/β diversity analysis revealed that Shannon, Ace, and Chao1 indices in R2 and R7 habitats were significantly higher than those in R1 and R8. Regarding community aggregation patterns, soil bacterial communities in R1, R9, and R12 habitats exhibited the highest aggregation, while those in R2 and R11 habitats showed relatively lower aggregation. Functional prediction demonstrated that Metabolism dominated across all distribution areas (50.40–52.02%), with the R11 habitat exhibiting exceptionally high metabolic function abundance (>9300). Clustering analysis partitioned the 12 habitats into two distinct groups: one comprising R2, R6, R7, R9, R10, and R11, and the other containing the remaining six habitats. Redundancy analysis (RDA) further clarified that habitat factors, including altitude, soil water content (SWC), east longitude (EAST), and pH, were key drivers shaping bacterial community structure. This study underscores the pivotal regulatory role of environmental factors in shaping rhizosphere microbial diversity, community structure, and functional profiles of Chinese seabuckthorn, thereby providing a valuable scientific foundation for the sustainable development of characteristic medicinal plant resources on the Qinghai–Tibet Plateau.

## Simple Summary

Exploring the compositional characteristics of bacteria in the rhizosphere soil of Chinese seabuckthorn across 12 regions on the Qinghai–Tibet Plateau and their relationships with the growth environment is of vital importance for facilitating the development of distinctive medicinal plant resources. By analyzing soil and climate data, as well as bacterial genetic information, we found that Proteobacteria, Acidobacteriota, and Actinobacteriota were the dominant bacterial phyla across all regions. Among them, Xunhua Salar Autonomous County exhibited the highest bacterial abundance, while both Xunhua Salar Autonomous County and Huangzhong District demonstrated relatively high bacterial Shannon diversity indices. In contrast, regions such as Ping’an District and Tongde County had more concentrated bacterial communities. The primary function of the bacteria was identified as Metabolism, with Yongdeng County showing particularly prominent metabolic activity. Furthermore, our research revealed that altitude, latitude and longitude, soil moisture content, and pH were key factors influencing bacterial distribution. These environmental factors regulated bacterial diversity, community structure, and functional traits. This study provides a crucial scientific basis for the rational development of medicinal plant resources on the Qinghai–Tibet Plateau and holds practical application value.

## 1. Introduction

*Hippophae rhamnoides* subsp. *sinensis* Rousi is a deciduous shrub or small tree belonging to the Elaeagnaceae family, commonly known as Chinese seabuckthorn, sour thorn, or black thorn [1,2,3,4,5]. This subspecies is primarily distributed in China, Nepal, and Bhutan, with its main production areas in China being Qinghai, Gansu, and Xinjiang provinces, while sporadic distributions occur in Hebei, Sichuan, Tibet, and other regions [1,2,3,4,5]. Chinese seabuckthorn holds significant ecological and economic value in the Qinghai–Tibet Plateau region, with its distribution range closely aligning with the geographical environmental characteristics of China’s Qinghai–Tibet Plateau [1]. Chinese seabuckthorn grows at an altitude of 1200 m~4800 m, and its growing environment is mostly found in sunny ridges, valleys, dry riverbeds or slopes, and gravelly or sandy soil or loess [2]. It has a variety of characteristics, such as cold tolerance, drought resistance, radiation resistance, and strong environmental adaptability, and is an ecological restoration plant with good stress resistance [3]. Meanwhile, Chinese seabuckthorn is a special medicinal resource plant in China, which is a Tibetan habitual medicinal herb [4]. According to the earliest existing classic of Tibetan medicine in China, *Yue Wang Yao Zhen*, Chinese seabuckthorn has remarkable effects of invigorating the spleen and aiding digestion, relieving cough and eliminating phlegm, promoting blood circulation, and removing blood stasis. In modern clinical applications, the fruit extract of Chinese seabuckthorn is mainly used to treat cardiovascular, digestive, respiratory, and immune systems, with characteristics such as lowering blood lipids, protecting gastric mucosa, anti-inflammatory and anti-asthmatic effects, and enhancing human immunity. It has high medicinal value and broad prospects for development and application [5].

Soil bacteria, as a crucial component of soil microorganisms, are extensively distributed in the rhizosphere soil of plants and form mutualistic symbiosis with plant hosts. Their diversity and structural functionality play pivotal roles in plant growth and developmental processes [6,7,8]. Rhizosphere soil bacteria can enhance plant adaptability to surrounding environments by modulating the soil microenvironment, nutrient cycling, and other ecosystem functions [6]. It has been shown that vegetation may affect the soil physicochemical properties and thus the soil bacterial community composition [6]. For example, planting mixed-sowing grasslands on the Qinghai–Tibet Plateau can decrease soil pH, increase the content of total nitrogen, total phosphorus, and total potassium in the soil, increase the relative abundance of Proteobacteria phylum and Actinobacteria phylum, and reduce the relative abundance of Acidobacteria phylumand Bacteroidetes phylum [7]. Concurrently, other studies have indicated that the composition and abundance of microbial communities can regulate plant growth and development, enhance plant resistance, and influence plant distribution patterns [8]. For example, nitrogen-fixing bacteria can convert airborne nitrogen into nitrogen-containing compounds in the soil, and these compounds can be used by plants to synthesize their own required amino acids or proteins as a way of fulfilling their own growth and development needs [8]. Furthermore, certain bacterial symbionts can elevate the activity of plant antioxidant protective enzymes and induce the production of corresponding secondary metabolites, enabling plants to resist adverse external environments and facilitating the expansion of their distribution ranges [9].

The Qinghai–Tibet Plateau has characteristics of high altitude, strong radiation, low oxygen content, a large temperature difference between day and night, and an obvious difference in water and heat distribution patterns. Plateau plants and rhizosphere soil bacteria build a complex and stable interaction system so that plants can better adapt to the unique climatic conditions of the Qinghai–Tibet Plateau [10,11]. In recent years, scholars at home and abroad have carried out research in many aspects of plant rhizosphere soil bacterial communities [12,13,14]. For example, Yang et al. [12] discovered, in their investigation of wild *Glyptostrobus pensilis* rhizosphere bacterial communities, that bacterial community structures varied with changes in soil nutrients and geoclimatic conditions, and that soil phosphorus content, potassium content, and moisture levels made significant contributions to bacterial community variation. Zhao et al. [13] evaluated the potential impacts of bacterial fertilizer on grapevine growth, development, and farmland soil properties by analyzing bacterial community diversity in grape rhizosphere soils. Lu et al. [14], through their research on soil microbial communities of *Phlomoides rotata*, revealed significant differences in soil microbial community structures and diversity across various distribution zones of this species on the Qinghai–Tibet Plateau, with organic carbon and total nitrogen content exerting significant regulatory effects on the distribution of rhizosphere microbial communities.

Current academic research on Chinese seabuckthorn predominantly focuses on the development of pharmacologically active components and ecological characteristics of natural populations, yet the structural and functional characteristics of rhizosphere bacterial communities in their wild populations remain systematically uncharacterized [1,2,3,4,5,15]. Targeting this knowledge gap, the present study selected 12 typical distribution areas on the Qinghai–Tibet Plateau as research sites, systematically collected rhizosphere soil samples of Chinese seabuckthorn, and integrated soil physicochemical indicators, regional climatic features, and high-throughput sequencing technology to elucidate the compositional characteristics, functional diversity, and coupling mechanisms with environmental factors of rhizosphere bacterial communities. The research proposed two core hypotheses: first, habitat heterogeneity shapes differentiated rhizosphere microenvironments by influencing host growth, driving adaptive differentiation of bacterial communities; second, significant synergistic response relationships exist between community characteristics and soil–climate factors. These findings will establish an “environment–host–microbe” interaction model, providing critical microbiological evidence for mining plant growth-promoting microbial resources and optimizing artificial cultivation systems, while advancing research on plant rhizosphere microbiomes in alpine ecological regions.

## 2. Materials and Methods

### 2.1. Overview of Chinese Seabuckthorn Sampling Sites

**Figure 1:**
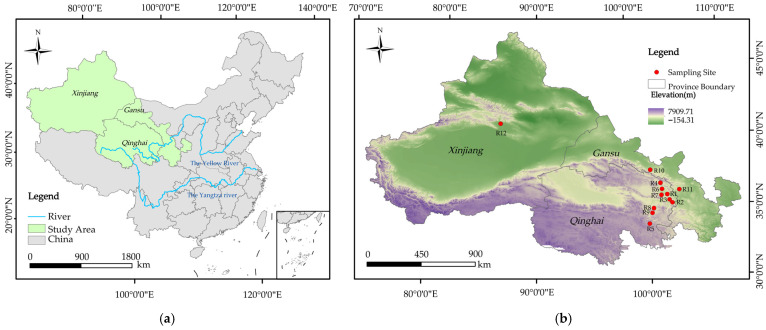
Sampling points of 12 Chinese seabuckthorn distribution areas in China. Note: (**a**) Map of China; (**b**) map of the study area (Gansu, Qinghai, and Xinjiang provinces). This figure was created using the standard map (approval number: GS (Beijing, China) No. 0650 (2024)) downloaded from the Standard Map Service Website of the China Bureau of Surveying and Mapping Geographic Information. The distance between sampling sites ranges from 34.28 to 15,817.74 km.

From mid-July to mid-August 2023, rhizosphere soil samples of Chinese seabuckthorn were collected from 12 regions across the Qinghai–Tibet Plateau, specifically including Ping’an District (R1), Xunhua Salar Autonomous County (R2), Hualong Huizu Autonomous County (R3), Menyuan Huizu Autonomous County (R4), Maqin County (R5), Datong Huzui and Tuzu Autonomous County (R6), Huangzhong District (R7), Guinan County (R8), and Tongde County (R9) in Qinghai Province; Minle County (R10) and Yongdeng County (R11) in Gansu Province; and Hejing County (R12) in the Xinjiang Uygur Autonomous Region. The geographical locations of these sampling sites are illustrated in Figure 1, while their detailed geographic coordinates and climatic characteristics are summarized in Supplementary Table S1.

All 12 sampling regions are characterized by a plateau continental climate, featuring low annual precipitation (30~140 mm), low annual average temperatures (−2.0~12.0 °C), extreme minimum temperatures reaching −25.5 °C, high annual average evaporation (1200~2500 mm), long annual average sunshine duration (2100~2800 h), and substantial diurnal temperature variations (up to 20~25 °C). Its companion plants usually include *Artemisia frigida*, *Elymus nutans*, *Poa annua*, *Saussurea arenaria*, *Astragalus polycladus*, *Lancea tibetica*, and *Gentiana crassicaulis*.

### 2.2. Rhizosphere Soil Sample Collection from Chinese Seabuckthorn

Within each of the 12 wild Chinese seabuckthorn natural populations, 9 replicate sampling units were systematically arranged along an S-shaped sampling transect, with a constant spacing of 8 m between adjacent units. We selected Chinese seabuckthorn plants during their peak fruiting stage, with tree ages ranging from 5 to 10 years. All selected sample plants belong to the Chinese seabuckthorn species and exhibit highly similar genotypes. However, due to differing habitats, populations show genetic variations including mutations, chromosomal recombination, and allelic differences, resulting in non-identical genotypes across populations. Variations in environmental factors and intrinsic genetic differences contribute to distinct growth patterns (plant morphologies) among the 12 populations. All sampled Chinese seabuckthorn plants have heights between 2.2 and 4.2 m, crown widths of 1.4–2.2 m, and ground diameters ranging from 3.5 to 5.5 cm. The rhizosphere soil of Chinese seabuckthorn was collected using the standardized Riley–Barber rhizosphere soil sampling method [16]. The operational procedure was as follows: First, the complete root system of Chinese seabuckthorn was excavated, followed by vertical shaking to naturally separate non-rhizosphere soil. Using a sterile brush, precise sampling was conducted along the root axis direction to systematically collect soil particles adhering to the root surface. Each sampling unit selected three adjacent sampling points, and equal amounts of rhizosphere soil samples were pooled. Through a combination design of 12 sampling areas × 3 replicate points, a total of 36 ecologically representative rhizosphere soil sample libraries were constructed.

All samples were collected from the vertical soil layer spanning a 0–50 cm depth. The rhizosphere soil samples were processed through a 2 mm aperture standard sieve and divided into two subsamples. Among them, the routine analysis samples were encapsulated in sterile bags and placed in −20 °C refrigerator for preservation; the microbiomics research samples were transferred to 50 mL centrifuge tubes, and after being briefly preserved in liquid nitrogen, they were transferred to a −80 °C ultra-low-temperature refrigerator (Zhongke duling, Anhui, China) for long-term preservation. The determination of soil physical and chemical indices strictly follows the specification of *Soil Agrochemical* Analysis [17] and measures pH, conductivity, water content, organic matter, and nitrogen, phosphorus, and potassium content. The specific measurement methods for the 10 soil indicators are as follows: Soil organic matter content (SOM) was determined using the potassium dichromate oxidation method; soil total nitrogen content (STN) was measured via the Kjeldahl digestion method; soil total phosphorus content (STP) was quantified using the molybdenum–antimony anti-colorimetric method; soil total potassium content (STK) was determined by flame photometry; soil available nitrogen (SAN), available phosphorus (SAP), and available potassium (SAK) content were measured using the alkali hydrolysis diffusion method, molybdenum–antimony anti-colorimetric method, and flame photometry, respectively; soil water content (SWC) was determined by the oven-drying method; soil pH and electrical conductivity (SEC) were measured using the potentiometric method and electrode method, respectively [1,17] (see Supplementary Table S2 for specific parameters). 

### 2.3. China Seabuckthorn Rhizosphere Soil Bacterial DNA Extraction, PCR Amplification, and High-Throughput Sequencing

For DNA extraction, 0.5 g of rhizosphere soil samples from Chinese seabuckthorn were utilized. The extracted DNA was subjected to 1% agarose gel electrophoresis, and its concentration and purity were evaluated using a nucleic acid analyzer (Illumina, San Diego, CA, USA) [18]. The samples were then sent to Beijing Novogene Bioinformatics Co., Ltd. (Beijing, China) for high-throughput sequencing, and the bacterial 16S rRNAV4 region was amplified using universal primer pairs 515F and 806R (515F: 5′-GTGCCAGCMGCCGCGGGTAA-3′; 806R: 5′-GGACTACHVGGGTWTCTAAT-3′) [19]. After the DNA sample amplification test was qualified, the mixing and purification of PCR products were carried out, and then the whole library preparation was completed through the steps of end repair, the addition of A-tail, the addition of sequencing connector, and purification. A small-fragment library was constructed according to the characteristics of the amplified region, and the MiSeq library was sequenced at both ends based on the Illumina MiSeq sequencing platform (Illumina, San Diego, CA, USA). After read splicing and filtering, amplicon sequence variants (ASVs) were denoised. Subsequent analyses involved species annotation and abundance profiling of the resulting high-quality data to elucidate the species composition of the samples [18,19]. Further visual analytics of soil microbial communities, including α-diversity, β-diversity, species composition, functional prediction, and clustering analyses, were conducted on the Novogene Cloud Platform (https://magic.novogene.com) (accessed on 5 May 2025). 

### 2.4. Data Analysis

Raw data organization and preprocessing were completed using Microsoft Excel 2019, and basic statistical analyses were performed with SPSS 25.0 software. Sequencing data denoising and feature extraction were processed using the DADA2 module of the QIIME (v2.0) platform, followed by the filtering out of low-quality sequences with abundance below 5, resulting in the generation of amplicon sequence variants (ASVs) and feature tables. Species annotation was performed by aligning ASVs with reference databases using the classify-sklearn module in QIIME2. Community diversity assessment involved generating rarefaction curves, relative abundance plots of species, PCoA plots, and UPGMA clustering trees based on the QIIME2 platform, and calculating 8 α-diversity indices for soil bacterial communities. Differences in community structure among samples were tested for significant differences in microbial community structure between groups using the LEfSe algorithm. Functional prediction was achieved by associating ASV annotation results with functional databases using PICRUSt software (v2.0) to predict the functional potential of microbial communities. Correlation heatmaps among environmental factors, soil physicochemical properties, and microbial diversity were visualized using the pheatmap package (version 1.0.12) in R 3.5.2 software. Constrained ordination analysis (RDA) was implemented using Canoco 5.0 software to analyze the associations between environmental factors and microbial communities.

## 3. Results

### 3.1. Sequencing Quality and Venn Analysis of Rhizosphere Soil Bacteria in Chinese Seabuckthorn

High-throughput 16S rRNA gene sequencing analysis was performed on 36 rhizosphere soil samples of Chinese seabuckthorn collected from 12 ecological regions in the Qinghai–Tibet Plateau (three replicates per region). Paired-end sequencing libraries were constructed using the Illumina MiSeq platform. As shown in Supplementary Figure S1a, the Shannon rarefaction curves demonstrate a notable plateauing trend (slope < 0.05) when sequencing depth reaches 10,000 reads. This indicates that the current sequencing strategy captured over 95% of the ASV diversity (Good’s Coverage > 99%), meeting the sequencing saturation criteria recommended by QIIME2. This result validates that the sequencing depth employed in this study adequately reflects the true species composition of the samples. As illustrated in Supplementary Figure S1b, the species accumulation curve shows that when the sample size reached 24, the new species discovery rate dropped below the 5% threshold. This meets the sampling saturation criteria recommended by ICMSF, with the curve entering a plateau phase, indicating sufficient sample size. The 36 samples adopted in this study (three replicates per group) far exceed this threshold, ensuring robust statistical power for community structure analysis. Through combined analysis of rarefaction curves (sequencing depth validation) and species accumulation curves (sample size validation), we demonstrate that the current experimental design achieves saturation in both dimensions. The integrated application of these curves provides scientific validation of the experimental design’s effectiveness. Therefore, the current dataset reliably reflects the authentic ecological characteristics of seabuckthorn rhizosphere bacterial communities in the Qinghai–Tibet Plateau.

By carrying out Venn analysis on the rhizosphere soil bacterial community of Chinese seabuckthorn in 12 types of habitats (Supplementary Figure S1c), it was found that there were significant differences in the distribution characteristics of amplicon sequence variants (ASVs) among the habitats. The R2 habitat exhibited the highest ASV richness (3496 ASVs), closely followed by the R7 habitat (2840 ASVs). In contrast, habitats R1 (1844 ASVs), R8 (2108 ASVs), R9 (2059 ASVs), and R12 (1925 ASVs) displayed relatively lower ASV counts, all below 2150 ASVs. Further analyses showed that a total of 151 ASVs were stably present in at least five types of habitats, and that the above shared ASVs constituted the core components of the rhizosphere soil bacterial community of Chinese seabuckthorn.

### 3.2. Abundance Changes in Bacterial Phyla and Genera in the Rhizosphere Soil of Chinese Seabuckthorn

**Figure 2:**
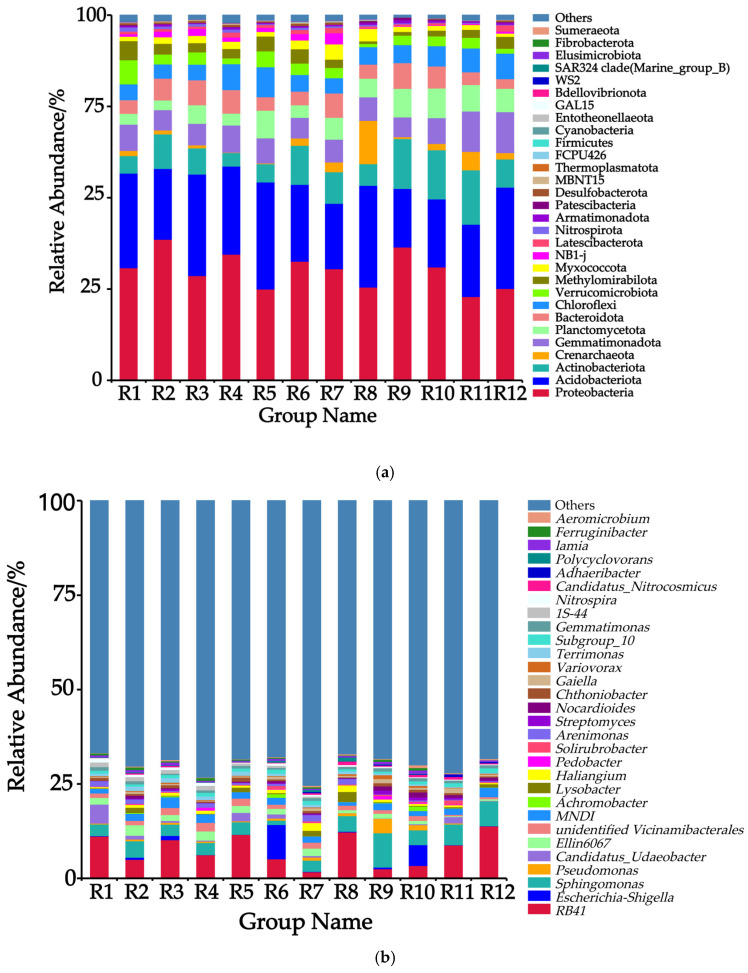
Relative abundances of 12 habitats at the level of phylum and genus. Note: (**a**) Phylum level; (**b**) genus level.

Among the rhizosphere soil samples of Chinese seabuckthorn from the 12 habitat types, the relative abundances of the top 30 bacterial phyla are presented in Figure 2a. At the phylum level, Proteobacteria phylum (22.93–38.61%), Acidobacteriota phylum (16.03–29.31%), Actinobacteriota phylum (3.69–14.89%), Crenarchaeota phylum (0.11–11.85%), and Gemmatimonadota phylum (5.43–11.21%) exhibited the highest relative abundances. Significant differences were observed in the ranking of bacterial phyla across the 12 habitats: Proteobacteria phylum constituted the dominant community in habitats R1, R2, R3, R4, R6, R7, R9, R10, and R11, while Acidobacteriota phylum predominated in habitats R5, R8, and R12. Compared to the R11 habitat, the other 11 habitats demonstrated elevated abundance of Proteobacteria phylum and reduced abundance of Actinobacteriota phylum in the soil bacterial communities.

Among the rhizosphere soil samples of Chinese seabuckthorn from the 12 habitat types, the relative abundances of the top 30 bacterial genera are presented in Figure 2b. At the genus level, *RB41* (Acidobacteria, 1.73–13.84%), *Escherichia-Shigella* (0.07–9.09%), *Sphingomonas* (1.15–9.09%), *Pseudomonas* (0.04–3.81%), and *Candidatus-Udaeobacter* (0.05–5.03%) exhibited the highest relative abundances across the 12 habitats. Significant differences were observed in the ranking of bacterial genera across the 12 habitats: *RB41* genus constituted the dominant genus in habitats R1, R2, R3, R4, R5, R8, R11, and R12; *Escherichia-Shigella* genus predominated in habitats R6 and R10; and *Sphingomonas* genus was dominant in habitats R7 and R9. Compared to the R12 habitat, the other 11 habitats demonstrated elevated abundances of the *Escherichia-Shigella* genus and the *Pseudomonas* genus, while the abundance of the *RB41* genus was reduced.

### 3.3. LEfSe Analysis of Rhizosphere Soil Bacteria in Chinese Seabuckthorn

As shown in Supplementary Figure S2a, through LEfSe analysis, the indicator species (LDA value over 4.0) with the most outstanding contribution to the difference in bacterial community in 12 kinds of habitats can be identified, and such analysis can clearly show the bacterial species that have a significant impact on the change in community structure. Combining the information from Supplementary Figure S2a,b, prominent indicator species were present in the bacterial communities of five of the twelve habitat types, and a total of six biomarkers were identified, one each for R1, R6, R8, and R12, and two each for R9. At the taxonomic level, the highest scoring biomarkers in habitats R1, R6, R8, R9, and R12 were *Candidatus-Udaeobacter* (4.36), *Escherichia-Shigella* (4.54), *Lysobacter* (4.11), *Sphingomonas* (4.51), and *RB41* (4.75) [Supplementary Figure S2b].

### 3.4. Diversity Changes in Rhizosphere Soil Bacteria of Chinese Seabuckthorn

#### 3.4.1. α-Diversity Changes in Rhizosphere Soil Bacteria of Chinese Seabuckthorn

**Figure 3:**
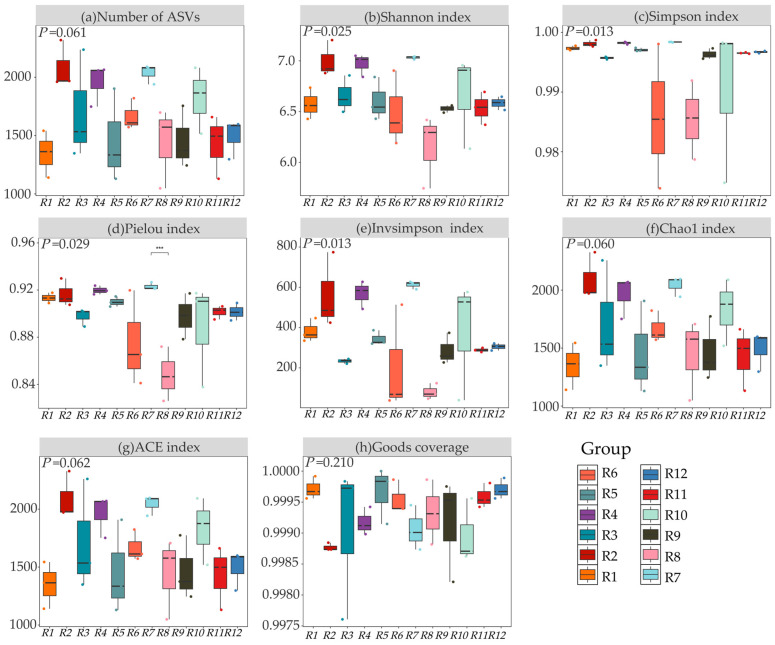
α-diversity of bacterial communities in 12 habitats. Note: (**a**) Number of ASVs, (**b**) Shannon index, (**c**) Simpson index, (**d**) Pielou index, (**e**) Invsimpson index, (**f**) Chao1 index, (**g**) ACE index, and (**h**) Goods coverage. *** represents *p* < 0.001.

As shown in Figure 3, significant differences (*p* < 0.05) were observed in the α-diversity of rhizosphere bacteria of Chinese seabuckthorn across 12 habitats. Specifically, the number of ASVs, the Ace index, and the Chao1 index in habitat R2 all reached their peaks (2082.33, 2092.12, and 2092.24, respectively), significantly higher than those in habitat R1 (with increases of 54.51%, 55.03%, and 55.00%, respectively). The Shannon index was highest in habitat R7 (7.03), significantly higher than that in habitat R8 (with an increase of 14.3%). Regarding the Simpson index, habitats R3, R10, R6, and R8 exhibited the lowest values, with most inter-habitat differences being non-significant (*p* > 0.05). The Pielou and Insimpson indices were also highest in habitat R7 (0.92 and 612.73, respectively), showing significant increases of 8.24% and 6.65-fold compared to habitat R8 (*p* < 0.05). The sequencing depth for all samples was ≥99.00%, ensuring data reliability. In summary, habitats R2 and R7 significantly enhanced bacterial ASV counts and diversity indices such as the Shannon, Ace, and Chao1 indices, outperforming habitats R1 and R8.

#### 3.4.2. Beta Diversity Changes in Rhizosphere Soil Bacteria of Chinese Seabuckthorn

**Figure 4:**
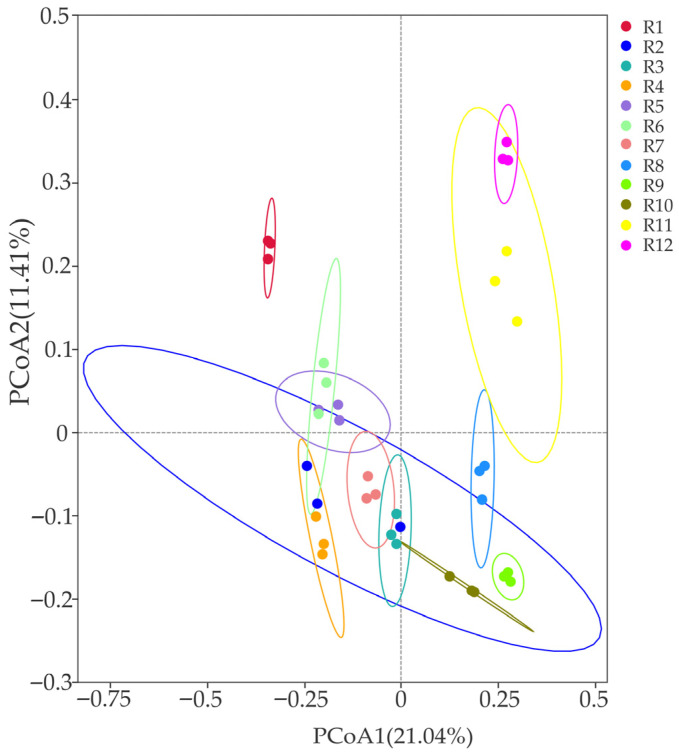
Differences in diversity of bacterial community β (PCoA) in 12 habitats.

The PCoA analysis based on Bray–Curtis distance (as shown in Figure 4) revealed that among the 12 habitats, the bacterial communities in habitats R2 and R11 exhibited significant within-group dispersion (with large compositional differences and low aggregation among samples). In contrast, the remaining 10 habitats demonstrated strong within-group homogeneity, particularly habitats R1, R9, and R12, which showed the highest similarity (with the smallest within-group coefficient of variation and high aggregation). The explanatory rates of PC1 and PC2 were 21.04% and 11.41%, respectively, accounting for a cumulative 32.45% and effectively reflecting differences in the community structure among habitats.

### 3.5. Co-Occurrence Network Analysis of Rhizosphere Soil Bacteria in Chinese Seabuckthorn

**Figure 5:**
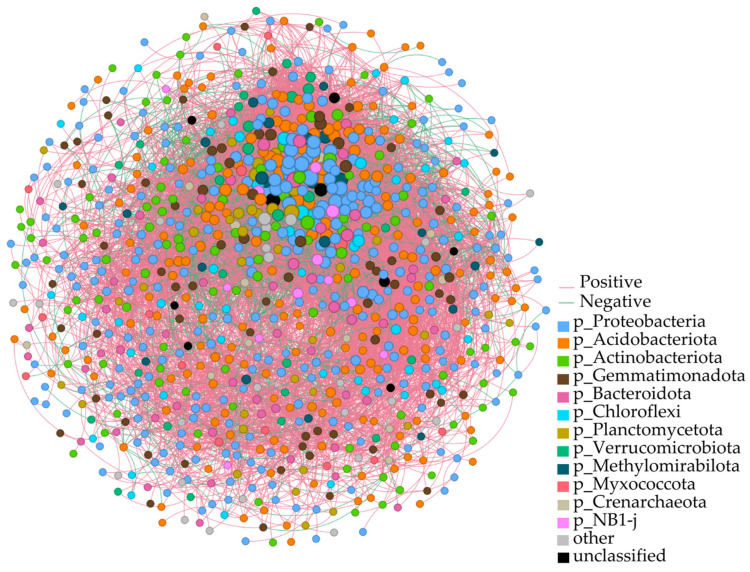
Molecular ecological network analysis of rhizosphere soil bacteria from Chinese seabuckthorn in 12 habitats. Note: Node color and size indicate bacterial phylum classification and ecological significance, respectively; edge color distinguishes interaction directions (red = positive correlation; green = negative correlation).

To investigate the co-occurrence patterns and inter-community interactions of bacterial phyla in the rhizosphere soils of Chinese seabuckthorn across 12 habitat types, a phylum-level co-occurrence molecular network was constructed (Figure 5). The results showed that the number of nodes at the level of rhizosphere soil bacterial phylum of Chinese seabuckthorn was 949, the number of edges was 15,703, the average degree was 33.09, the average path length was 1.92, the diameter of the network was 5.61, the density of the network was 0.03, the clustering coefficient was 0.44, and 70.85% of the correlated edges were positively correlated. Among the bacterial phyla, Proteobacteria (32.66%), Acidobacteriota (21.81%), Actinobacteriota (10.96%), Gemmatimonadota (7.23%), Bacteroidota (5.96%), and Chloroflexi (5.00%) exhibited high connectivity, centrality, and abundance. These phyla occupied central positions within the network, demonstrating significant influence through extensive interactions with other bacterial phyla.

### 3.6. PICRUSt Functional Analysis of Rhizosphere Soil Bacteria in Chinese Seabuckthorn

**Figure 6:**
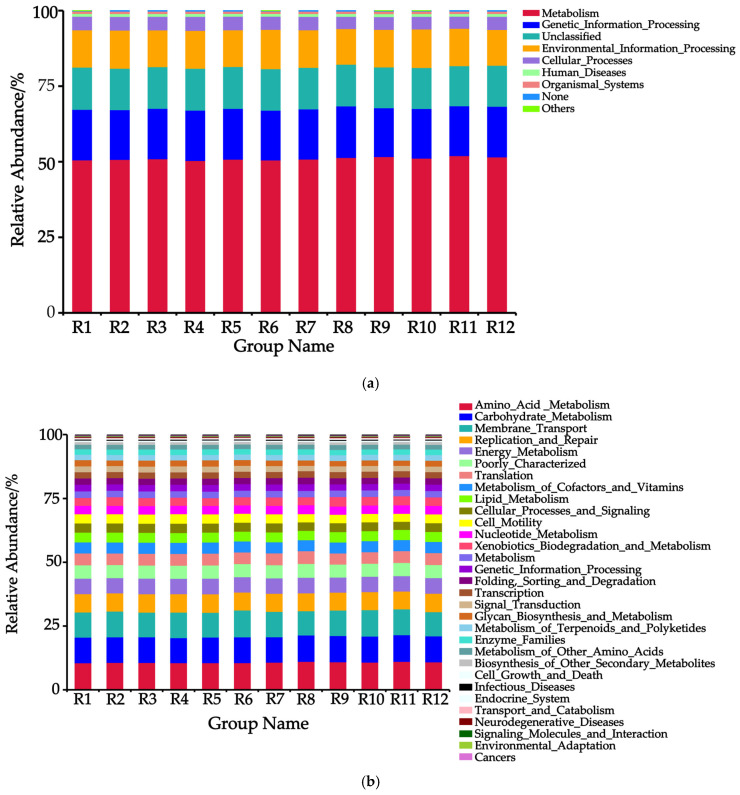

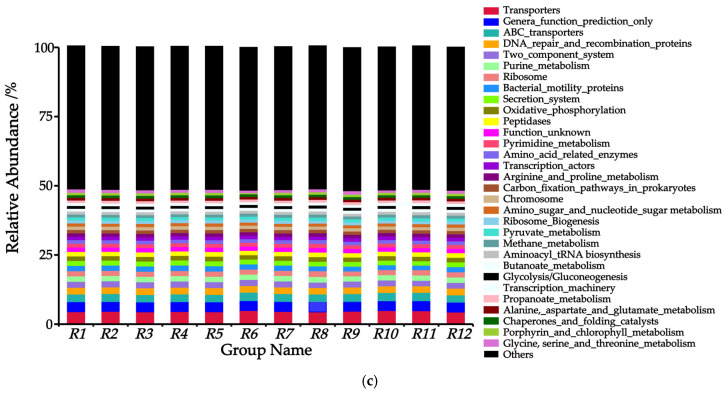
Predicted relative abundance of soil bacterial PICRUSt functions in 12 habitats. Note: (**a**) Level 1, (**b**) Level 2, and (**c**) Level 3.

Based on the PICRUSt Level 1 functional classification (Figure 6a), a total of eight functional types were identified, with the top five being Metabolism, Genetic Information Processing, Unclassified, Environmental Information Processing, and Cellular Processes in descending order. Among these, Metabolism functions dominated across all habitats, with the highest abundance observed in the R11 habitat (52.02%) and the lowest in the R4 habitat (50.40%). Compared to the R8 habitat, the other 11 habitats exhibited a decrease in the abundance of Genetic Information Processing and an increase in the abundance of Environmental Information Processing.

Based on the PICRUSt Level 2 functional classification (Figure 6b), a total of 31 functional modules were identified, among which 13 core modules (with abundance > 75%) included Amino_Acid_Metabolism, Carbohydrate_Metabolism, Membrane_Transport, Replication_and_Repair, Energy_Metabolism, Poorly_Characterized, Translation, Metabolism_of_Cofactors_and_Vitamins, Lipid_Metabolism, Cellular_Processes_and_Signaling, Cell_Motility, Nucleotide_Metabolism, and Xenobiotics_Biodegradation_and_Metabolism. The dominant modules were Amino_Acid_Metabolism (10.55–11.12%), Carbohydrate_Metabolism (9.81–10.44%), and Membrane_Transport (9.48–10.54%), with their combined abundance exceeding 25%. Notably, compared to the R8 distribution area, the other 11 regions exhibited significantly lower bacterial abundances in the functional modules of Amino_Acid_Metabolism, Poorly_Characterized, Translation, and Nucleotide_Metabolism, while showing higher bacterial abundances in the modules of Replication_and_Repair, Metabolism_of_Cofactors_and_Vitamins, and Lipid_Metabolism.

Based on the PICRUSt Level 3 functional classification (Figure 6c), a total of 31 functional modules were identified, among which five core modules (with relative abundance > 15%) included Transporters, Genera_function_prediction_only, ABC_transporters, DNA_repair_and_recombination_proteins, and Two_component_system. Notably, Transporters dominated across all habitats, with the highest abundance observed in the R6 habitat (4.92%) and the lowest in the R12 habitat (4.37%). Compared to the R8 habitat, the other 11 habitats exhibited a decrease in the abundance of General_function_prediction_only, while showing an increase in the abundances of DNA_repair_and_recombination_proteins and Two_component_system.

### 3.7. UPGMA Clustering of Rhizosphere Soil Bacteria in Chinese Seabuckthorn

UPGMA clustering analysis was conducted on the rhizosphere soil bacterial samples from 12 habitats of Chinese seabuckthorn (based on the Bray–Curtis distance matrix of ASVs, integrating the abundances of the top 20 phyla-level species). The results (Supplementary Figure S3) revealed that at a clustering distance of 0.192, the 12 habitats were divided into two groups—Group 1 (R2, R6, R7, R9, R10, and R11) and Group 2 (R1, R3, R4, R5, R8, and R12); Group 1, at a distance of 0.186, was further divided into a subgroup containing R11 and another subgroup containing the remaining five habitats, while Group 2, at a distance of 0.174, was divided into a subgroup containing R8 and another subgroup containing the remaining five habitats.

### 3.8. Coupling Relationship Between Rhizosphere Soil Bacterial Communities and Habitat Factors in Chinese Seabuckthorn

#### 3.8.1. Mantel Test Analysis Between Bacterial Community Structure and Habitat Factors

**Figure 7:**
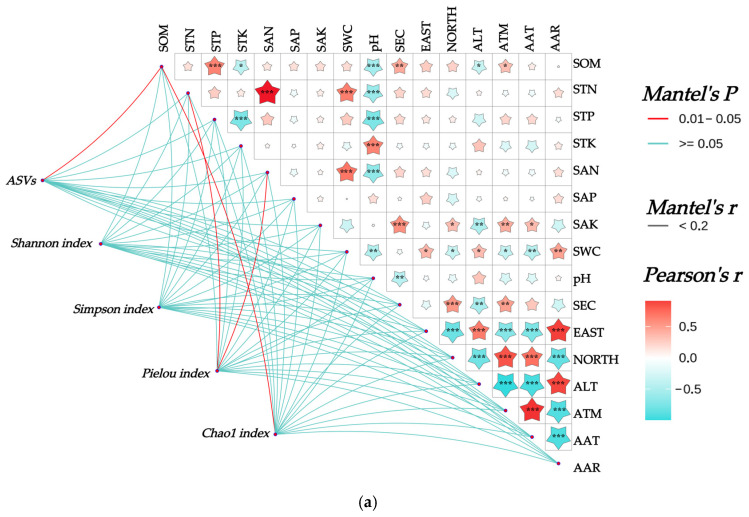

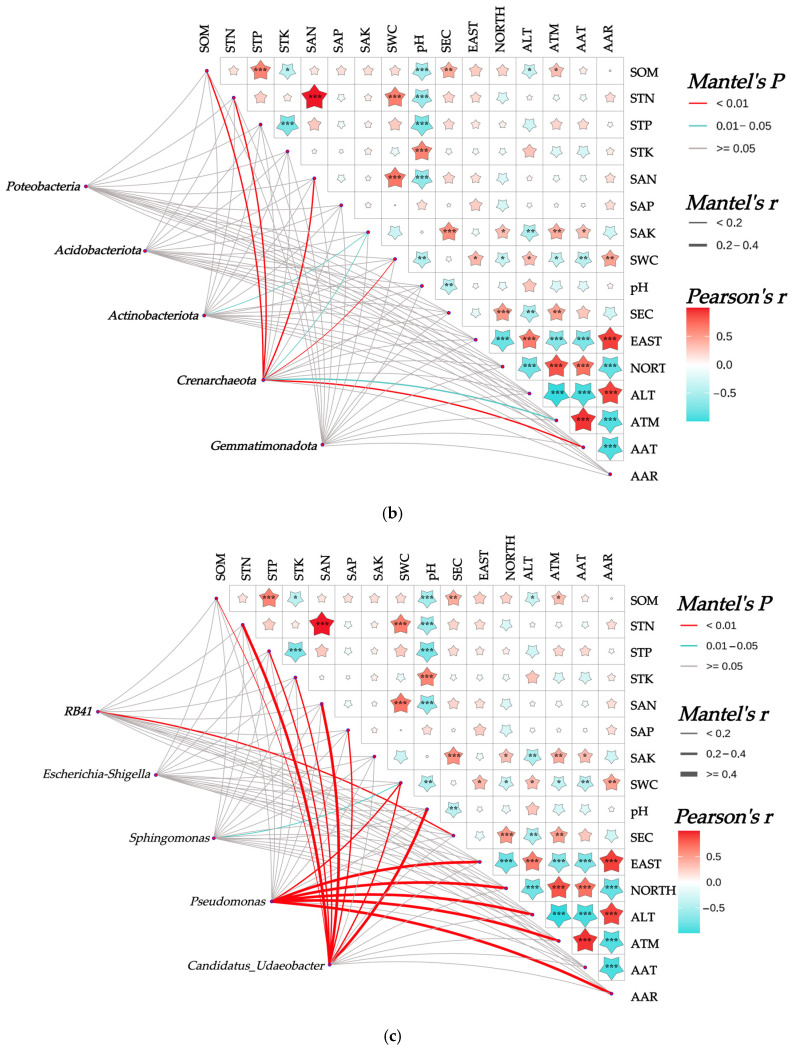
Mantel analysis between rhizosphere soil bacterial community and habitat factors of Chinese seabuckthorn. Note: * denotes *p* < 0.05, ** denotes *p* < 0.01, and *** denotes *p* < 0.001. (**a**) α-diversity, (**b**) bacterial phylum level, and (**c**) bacterial genus level.

The Mantel test revealed (Figure 7) the following highly significant correlations (*p* < 0.001) among the 16 soil–climate indicators: SOM exhibited a highly significant positive correlation with STP and a highly significant negative correlation with pH. STN showed highly significant positive correlations with SAN and SWC, while demonstrating a highly significant negative correlation with pH. STP displayed highly significant negative correlations with both pH and STK. STK had a highly significant positive correlation with pH. SAN exhibited a highly significant positive correlation with SWC and a highly significant negative correlation with pH. SAK showed a highly significant positive correlation with SEC; SEC demonstrated highly significant positive correlations with NORTH; EAST showed a highly significant positive correlation with AAR and ALT; EAST displayed highly significant negative correlations with AAT, ATM, and NORTH. NORTH showed highly significant positive correlations with AAT and ATM, while exhibiting highly significant negative correlations with AAR and ALT. ALT had a highly significant positive correlation with AAR and highly significant negative correlations with AAT and ATM. ATM exhibited a highly significant positive correlation with AAT and a highly significant negative correlation with AAR. AAT demonstrated a highly significant negative correlation with AAR.

The correlations between the rhizosphere soil bacterial community of Chinese seabuckthorn and habitat factors are illustrated in Figure 7. At the α-diversity level (Figure 7a), the number of ASVs and the Chao 1 index both exhibited significant correlations with SOM (*p* < 0.05). The Pielou index demonstrated significant correlations with STN and SAN (*p* < 0.05). In contrast, neither the Shannon nor Simpson indices showed significant associations with any of the 10 soil physicochemical properties or 6 environmental factors. At the phylum level (Figure 7b), the abundance of Actinobacteriota was significantly correlated with SAK (*p* < 0.05). The abundance of Crenarchaeota displayed highly significant correlations with SOM, STN, SAN, SWC, and AAT (*p* < 0.01), as well as significant correlations with SAK and ATM (*p* < 0.05). Proteobacteria, Acidobacteriota, and Gemmatimonadota showed no significant correlations with any soil physicochemical properties or environmental factors. At the genus level (Figure 7c), *RB41* exhibited a highly significant correlation with SEC (*p* < 0.01), while *Sphingomonas* demonstrated a significant correlation with SWC (*p* < 0.05). *Pseudomonas* was highly significantly correlated with SWC, EAST, NORTH, ALT, ATM, and AAR (*p* < 0.01). *Candidatus-Udaeobacter* showed highly significant correlations with SOM, STN, STP, STK, SAN, SAP, SWC, and pH (*p* < 0.01). *Escherichia-Shigella* exhibited no significant correlations with any of the indicators.

#### 3.8.2. RDA Between Bacterial Community Structure and Habitat Factors

**Figure 8:**
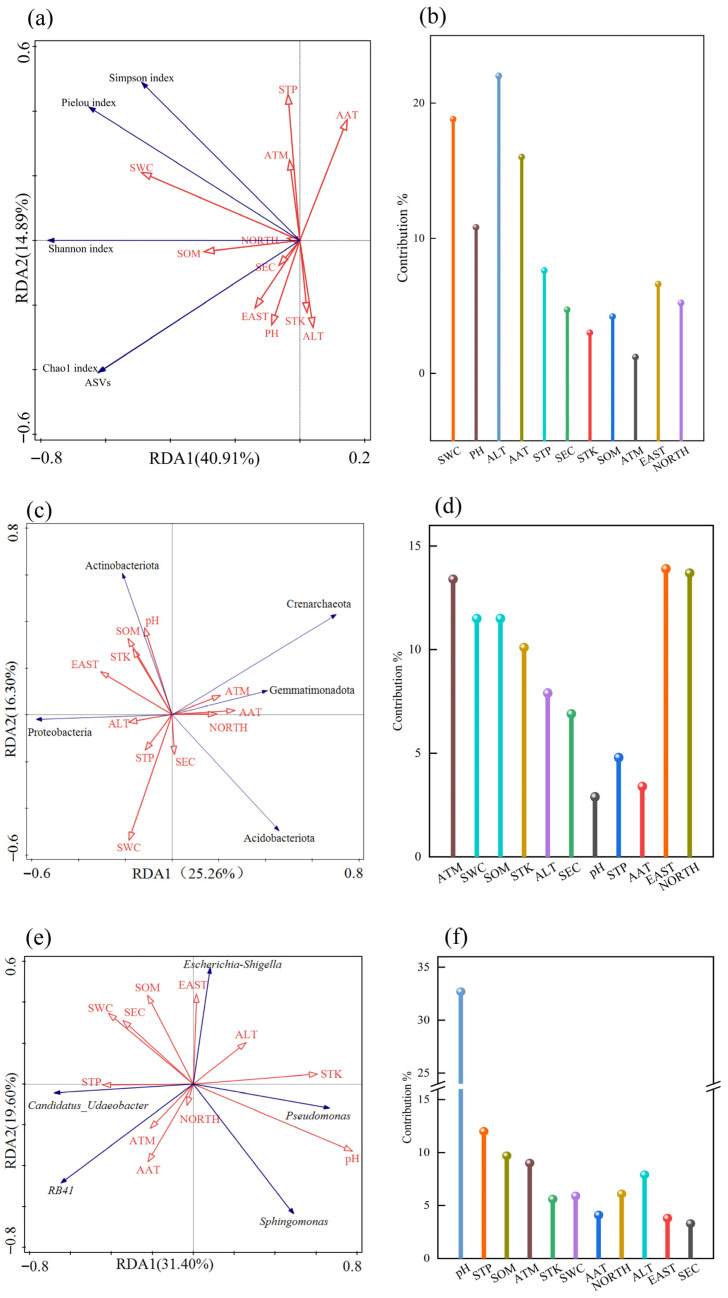
RDA between the rhizosphere soil bacterial community structure and habitat factors of Chinese seabuckthorn. Note: Red arrows indicate plant community characteristics and soil factors, and blue arrows indicate fungal diversity indices. (**a**,**b**) α-diversity; (**c**,**d**) bacterial phylum level; (**e**,**f**) bacterial genus level.

Based on the redundancy analysis (RDA) presented in Figure 8, the associations between the α-diversity of rhizosphere soil bacterial communities, the abundance of dominant phyla/genera, and habitat factors in Chinese seabuckthorn were revealed. Specifically, Figure 8a,b focus on the linear constraint relationships between α-diversity and environmental factors. Soil bacterial community diversity with climatic characteristics and soil physicochemical characteristics explained 40.91% and 14.89% in axes I and II, respectively, with a cumulative explanation of up to 55.80%. Among the environmental variables, altitude (ALT) exhibited the longest arrow line, with an explanation rate of 12.4% and a contribution rate of 22.0%, reaching a significant level (*p* = 0.010). Followed by soil water content (SWC), the explanation rate was 10.6%, the contribution rate was 18.8%, and the *p* value was 0.028, reaching a significant level. These findings indicate that altitude (ALT) is the most influential environmental factor shaping the rhizosphere soil bacterial community diversity of Chinese seabuckthorn, followed by soil water content (SWC).

Based on Figure 8c,d, the redundancy analysis (RDA) results for the top five bacterial phyla (by abundance) in the rhizosphere soil of Chinese seabuckthorn and habitat factors are presented. The model explained 25.26% and 16.30% of the variance along the first and second axes, respectively, with a cumulative explained variance of 41.56%. The environmental factor screening revealed that EAST exhibited the longest environmental vector arrow, accounting for 8.0% of the variance individually (contribution rate of 13.9%, *p* = 0.008), and served as the primary driver of phylum-level abundance variations. NORTH and ATM explained 7.9% and 7.7% of the variance, respectively (contribution rates of 13.7% and 13.4%; *p* = 0.012 and *p* = 0.042), forming secondary key influencing factors. Based on Figure 8e,f, the redundancy analysis (RDA) results for the top five bacterial genera (by abundance) in the rhizosphere soil of Chinese seabuckthorn and habitat factors are presented. The model explained 31.40% and 19.60% of the variance along the first and second axes, respectively, with a cumulative explained variance of 51.00%. Environmental factor screening revealed that soil pH exhibited the longest environmental vector arrow, accounting for 23.0% of the variance individually (contribution rate of 32.7%, *p* = 0.002), and served as the primary driver of genus-level abundance variations. The STP explained 8.5% of the variance (contribution rate 12.0%, *p* = 0.008), indicating secondary influence potential. The study confirmed that among multiple environmental factors, EAST, NORTH, ATM, soil pH, and STN collectively shaped the phylum- and genus-level abundance patterns of rhizosphere bacterial communities through synergistic effects of climatic and soil physicochemical factors.

## 4. Discussion

### 4.1. Compositional Structural Variation Characteristics of Rhizosphere Soil Bacterial Communities in Chinese Seabuckthorn Across Twelve Different Habitats

Plant rhizosphere bacteria, as key drivers of soil ecosystem functioning, profoundly influence plant growth and development through mechanisms including organic matter decomposition, nutrient cycling facilitation, and pathogenic microorganism suppression [20,21]. This study focused on 12 wild germplasm distribution areas of Chinese seabuckthorn on the Qinghai–Tibet Plateau, systematically collecting rhizosphere soil samples and integrating physicochemical parameter analysis with high-throughput sequencing technology to elucidate the characteristics of rhizosphere bacterial communities and their environmental adaptation mechanisms. The results revealed significant variations in soil physicochemical properties and geoclimatic conditions across different habitats, directly influencing Chinese seabuckthorn plant growth and root development, which subsequently shaped bacterial community composition and structure. Specifically, habitats R2 (3496 ASVs) and R7 (2840 ASVs) exhibited the highest bacterial ASV counts, significantly exceeding that of the acidic R1 habitat (1844 ASVs). This aligns with Chinese seabuckthorn’s preference for weakly alkaline environments, where neutral-alkaline conditions may enhance plant–microbe symbiosis to amplify bacterial populations [22]. Conversely, the number of amplicon sequence variants (ASVs) was lower in high-altitude habitats R8 (2108 ASVs) and R9 (2059 ASVs) (at elevations exceeding 3300 m). This confirms that as altitude increases, atmospheric radiation intensifies, while temperature and air pressure decrease, which in turn impairs plant growth, inhibits the decomposition of soil litter, and reduces soil bacterial abundance [23]. Further research revealed that the ecological niche width of the rhizosphere bacterial community of Chinese seabuckthorn exhibited a significant “higher in the middle, lower at both ends” distribution pattern along the altitudinal gradient. Specifically, it peaked in mid-altitude regions while significantly narrowing in extreme environments at both high and low altitudes. The underlying reason for this phenomenon may be that, as a pioneer nitrogen-fixing plant, Chinese seabuckthorn actively shapes its rhizosphere microenvironment through root exudates in mid-altitude zones. This not only buffers environmental filtering pressures but also facilitates the recruitment of microbial groups with specific functions (such as nitrogen fixation, acid tolerance, and drought tolerance), thereby achieving adaptive expansion of its ecological niche in mid-altitude regions.

Further analysis revealed that bacterial communities across the 12 habitats exhibited similarities in species composition. At the phylum level, Proteobacteria, Actinobacteria, Acidobacteriota, Gemmatimonadota, and Crenarchaeota constituted the top five dominant phyla by abundance. These findings are consistent with Liu et al.’s [24] research on dominant rhizosphere bacterial phyla of seabuckthorn, indicating that these phyla play pivotal ecological roles in the rhizosphere environment of Chinese seabuckthorn. Notably, significant inter-habitat differences in species composition were observed, likely associated with the synergistic selection effects of environmental factors such as SWC. For instance, the R7 habitat with higher SWC exhibited a marked increase in the relative abundance of *Sphingomonas*, confirming the positive correlation between its abundance and soil water content [25,26]. This finding further confirms that habitat climatic features and soil conditions are key factors influencing plant growth and root development, while driving the differentiation of dominant bacterial genera in rhizosphere communities [26].

This study demonstrates significant variations in the diversity of rhizosphere soil bacteria associated with Chinese seabuckthorn in 12 habitats on the Qinghai–Tibet Plateau. Specifically, the bacterial Shannon index, Ace index, Chao 1 index, and the number of ASVs were significantly higher in the remaining 10 habitats, especially the R2 and R7 habitats, compared to the R1 and R8 habitats. These findings align with previous studies by Zhang et al. [27] and Lou et al. [28], underscoring the pronounced influence of habitat on soil bacterial diversity. This influence is likely attributable to variations in soil physicochemical properties, such as pH, water content, and nutrient availability, as well as climatic characteristics. PCoA analyses revealed that the compositional structure of soil bacteria from different habitats had different characteristics. Habitats R1, R9, and R12 displayed relatively high aggregation, whereas the bacterial communities in the other nine habitats exhibited greater dispersion. Notably, the rhizosphere microbial community structure of Chinese seabuckthorn in habitat R12 diverged significantly from that in other habitats. It is hypothesized that such differences in community structure may be associated with variations in soil factors between habitats, and heterogeneity in soil matrix properties may be a central driver of bacterial community compositional divergence [29].

The rhizosphere microbial ecological network map can effectively respond to the interactions between plant rhizosphere microorganisms and the stability of bacterial communities [30,31]. In this study, microbial co-occurrence network analysis was employed to predict core taxa within the bacterial community, which play pivotal roles in shaping network structures and ecological functions of plant rhizosphere soil systems. These core taxa contribute to critical processes such as pathogen suppression in agricultural settings [30] and facilitating microbial synergies that enhance plant stress resistance [31]. Our findings reveal a highly complex bacterial network in the rhizosphere of Chinese seabuckthorn, characterized by 949 nodes and 15,703 edges, with 70.85% of interactions exhibiting positive correlations. This indicated that most of the coexisting bacteria in the inter-root soil of Chinese seabuckthorn were in a cooperative relationship, such as mutual benefit, interactive symbiosis, and cohabitation, and that the structure of this bacterial community was conducive to the acquisition of nutrients by the root system of the plant. Furthermore, the analysis highlights Proteobacteria phylum (32.66%), Acidobacteriota phylum (21.81%), and Actinobacteriota phylum (10.96%) as the most centrally connected, abundant, and topologically pivotal phyla within the network. These phyla emerge as the most influential bacterial groups in the 12 Qinghai–Tibet Plateau habitats, playing critical roles in maintaining the structural and functional stability of rhizosphere bacterial communities, improving soil conditions, and enhancing plant productivity in this alpine ecosystem [32].

Clustering analysis revealed that the 12 Chinese seabuckthorn rhizosphere bacterial communities were partitioned into two distinct groups: Group 1, comprising R2, R6, R7, R9, R10, and R11 habitats, and Group 2, containing the remaining habitats. This grouping pattern may be associated with differences in vegetation–soil characteristics across habitats, while climatic factors could also exert influence [1,33]. Integrating PICRUSt functional prediction analysis revealed significant functional differentiation in soil bacterial communities across the 12 habitats, consistent with Wang et al.’s [34] conclusion that bacterial community structure drives functional disparities. Specifically, Metabolism and Genetic Information Processing dominated across all habitats—likely a consequence of bacterial vital activities and evolutionary selection—where metabolic functions ensure energy and material supply, while genetic information processing drives adaptation and innovation. Their synergistic interaction confers core ecological status to Chinese seabuckthorn rhizosphere bacteria within soil ecosystems [35,36]. Notably, the Environmental Information Processing function in the R8 habitat was significantly weaker than in other habitats. This was likely attributed to its extreme elevation (3522 m) coupled with low SOM, STN, STP, and SWC. The barren and arid soil habitat may impose multiple stressors—including toxic effects and resource limitations—on Chinese seabuckthorn plants, inhibiting root growth and plant development. Consequently, these conditions suppress the growth and reproduction of rhizosphere bacterial communities, ultimately constraining bacterial Environmental Information Processing capabilities [37].

### 4.2. Coupling Relationship Between Rhizosphere Soil Bacterial Community Structure and Habitat Factors in Chinese Seabuckthorn

Mantel test analyses showed significant correlations between the physicochemical properties (organic matter, total nitrogen, total phosphorus, total potassium, pH, and electrical conductivity) of rhizosphere soils of Chinese seabuckthorn and the climatic characteristics of the habitat (longitude, latitude, altitude, precipitation, and temperature). Significant negative correlations were observed between soil organic matter (SOM), soil total nitrogen (STN), soil total phosphorus (STP), and pH, which may be attributed to the inhibitory effects of alkaline conditions on organic matter decomposition and nitrogen and phosphorus mineralization processes [38]. Conversely, there was a highly significant positive correlation between STN, SAN, and SWC, and it was hypothesized that this phenomenon may be related to microbial metabolism, and that moderately wet environments promote microbial reproduction and enzyme activity to accelerate the conversion of organic matter decomposition to inorganic nitrogen [39]. A significant negative correlation between SOM and altitude (ALT) was also identified, potentially resulting from dual constraints of microbial activity and vegetation growth. Specifically, excessive ALT may suppress both rhizosphere microbial activity and the physiological performance of Chinese seabuckthorn, leading to reduced soil organic matter accumulation [23]. Further analysis revealed significant correlations (*p* < 0.05) between bacterial community diversity, phylum/genus-level abundance composition, and habitat factors. Specifically, the Pielou evenness index, Crenarchaeota phylum abundance, and *Candidatus-Udaeobacter* genus abundance demonstrated significant (*p* < 0.05)/highly significant (*p* < 0.01) correlations with soil physicochemical properties (SOM, STN, SAN) and climatic features (EAST, NORTH, ATM, AAT). This indicates that through the synergistic interaction of “vegetation–habitat–microorganisms,” the Chinese seabuckthorn populations on the Qinghai–Tibet Plateau achieve both an enhancement in soil fertility and an optimization of the rhizosphere microbial community structure [40,41].

Soil physicochemical properties and climatic characteristics exert a significant shaping effect on the structure and diversity of rhizosphere bacterial communities of Chinese seabuckthorn on the Qinghai–Tibet Plateau, with altitude (ALT) emerging as a key regulatory factor. Studies have shown that altitude regulates the rhizosphere microbial community of Chinese seabuckthorn through three major mechanisms [23,29,42]: (1) An increase in altitude leads to enhanced radiation, decreased temperature, reduced air pressure, and diminished precipitation, directly inhibiting bacterial metabolic activity (enzyme activity) as well as growth and reproduction, thereby reducing microbial community diversity [23]. (2) Elevated altitude affects temperature, radiation, and air pressure, triggering a decline in vegetation productivity, a reduction in litter input, and a slowdown in decomposition rates, resulting in a significant decrease in soil total organic carbon, total nitrogen, and total phosphorus content, which indirectly suppresses soil bacterial growth [29]. (3) In high-altitude areas, the reduction in plant species leads to a simplification of root exudates, weakening plant–microbe interactions and causing a sharp decline in the number of relevant symbiotic bacterial species [42]. This process indicates that altitude not only directly influences the rhizosphere soil microbial community of Chinese seabuckthorn through ultraviolet radiation, precipitation, temperature, air pressure, and soil fertility but also indirectly regulates microbial community stability via the feedback mechanism of the habitat factor–plant–microbe interaction network [23,29,42]. The mechanism of soil water content (SWC) on the diversity of bacterial communities in Chinese seabuckthorn rhizosphere soils was second only to altitude, which shaped the bacterial community structure through direct water stress, in agreement with the results of Li et al. [43]. Among other environmental factors, EAST, NORTH, ATM, soil pH, and STN can affect the root growth and plant development of Chinese seabuckthorn through the synergistic effect of climate and soil physical and chemical factors, and then shape the abundance structure of the rhizosphere bacteria community of Chinese seabuckthorn.

## 5. Conclusions

This study systematically revealed the structural and functional characteristics of rhizosphere soil bacterial communities of Chinese seabuckthorn on the Qinghai–Tibet Plateau and their driving factors. Key findings include the following: (1) Among the 12 habitats, the R2 and R7 habitats exhibited significantly higher bacterial ASV counts (reaching 3496 and 2840, respectively) and certain α-diversity indices (Shannon index, Ace index, and Chao1 index) compared to R1, R8, R9, and R12 habitats. (2) Proteobacteria, Acidobacteriota, Actinobacteriota, Crenarchaeota, and Gemmatimonadota were identified as dominant taxa in the rhizosphere bacterial communities. Notably, Proteobacteria maintained a relative abundance exceeding 20% across all habitats, demonstrating strong adaptability to alpine extreme environments. (3) Functional predictions revealed the dominance of Metabolism and Genetic Information Processing functions. The R8 habitat showed significant enrichment of Genetic Information Processing but lower abundance of Environmental Information Processing. (4) Bray–Curtis clustering analysis grouped the 12 habitats into two major clusters: one comprising R2, R6, R7, R9, R10, and R11, and the other containing the remaining habitats. Redundancy analysis (RDA) demonstrated that altitude (ALT), soil water content (SWC), east longitude (EAST), and pH jointly shaped the α-diversity and phylum/genus-level abundance patterns of rhizosphere bacterial communities through synergistic interactions between climatic and soil physicochemical factors.

In summary, habitat heterogeneity shapes differentiated rhizosphere microenvironments by influencing the growth of Chinese seabuckthorn, thereby driving adaptive differentiation of bacterial communities. Concurrently, significant synergistic responses exist between the characteristics of Chinese seabuckthorn’s rhizosphere bacterial communities and soil–climate factors. Future research will focus on the isolation and identification of rhizosphere and endophytic bacteria from Chinese seabuckthorn, conduct inoculation experiments to verify plant growth-promoting effects, integrate metabolomics to analyze changes in the medicinal properties of Chinese seabuckthorn, and assess the applicability of these bacterial strains to other alpine plants.

## Data Availability

The data supporting the findings of this study are available in the NCBI epository under accession number PRJNA1310069 (https://www.ncbi.nlm.nih.gov/sra/PRJNA1310069), (accessed on 24 August 2025). For further inquiries, please contact the corresponding author.

## Supplementary Materials

The following supporting information can be downloaded at: https://www.mdpi.com/article/10.3390/biology14091304/s1, Figure S1: Analysis of sequencing results quality and ASVs quantity of 12 habitats; Figure S2: LEfSe analysis of 12 habitats at genus level; Figure S3: UPGMA clustering tree based on weighted Bray-Curtis distance; Table S1: Geographical and climatic conditions of the growth regions of Chinese seabuckthorn; Table S2: Physicochemical properties of soils in Chinese seabuckthorn distribution areas.
