# Supplementary material for: The Bacterial Community Characteristics of Hippophae rhamnoides Subsp. sinensis Rousi in Different Distribution Areas of the Qinghai–Tibet Plateau and Their Responses to Habitat Factors
Source: Biology (Basel). 2025 Sep 21;14(9):1304. doi: 10.3390/biology14091304 (PMC12467999; doi:10.3390/biology14091304)
Supplement: Supplementary file 1 [file biology-14-01304-s001.zip › biology-3867948-supplementary.pdf]

biology

Supplementary Material

1 Supplementary Table

Supplementary Table S1. Geographical and climatic conditions of the growth regions of Chinese seabuckthorn. **Note:** EAST: East longitude; NORTH: Northern latitude.

Serial number	Sampling location	EAST /°	NORTH /°	Altitude（ALT/m）	Air pressure（ATM/KPa）	Average annual temperature（AAT/℃）	Average annual rainfal（AAR/mm）
R1	Ping'an District	102.11	36.50	2497.83	73.83	3.78	114.9
R2	Xunhua Salar Autonomous	102.49	35.85	2610.68	74.07	2.38	121.89
R3	Hualong Huizu Autonomous	102.26	36.09	2503.98	73.09	3.67	114.75
R4	Menyuan Huizu Autonomous	101.61	37.39	3369.01	67.98	−1.77	134.44
R5	Maqin County	100.24	34.48	3353.58	67.94	0.62	125.88
R6	Datong Huzui and Tuzu	101.69	36.93	2748.76	73.24	2.60	125.64
R7	Huangzhong District	101.57	36.50	2906.80	71.94	1.86	124.36
R8	Guinan County	100.75	35.59	3522.05	66.80	0.25	120.74
R9	Tongde County	100.58	35.25	3369.01	67.94	1.47	124.87
R10	Minle County	100.81	38.43	2692.50	73.17	0.88	116.38
R11	Yongdeng	103.26	36.74	2214.63	77.87	4.39	101.47
R12	Hejing County	86.38	42.32	1106.30	87.81	10.76	31.56

Supplementary Table S2. Physicochemical properties of soils in Chinese seabuckthorn distribution areas.**Note:** SOM: Soil organic matter; STN: Soil total nitrogen; STP: Soil total phosphorus; STK: Soil total potassium; SAN: Soil alkali-hydrolyzable nitrogen; SAP: Soil available phosphorus; SAK: oil available potassium; SWC: Soil water content; SEC: Soil electrical conductivity.

Serial number	SOM/ (g/kg)	STN/ (g/kg)	STP/ (g/kg)	STK/ (g/kg)	SAN/ (mg/kg)	SAP/ (mg/kg)	SAK/ (mg/kg)	SWC/ (%)	pH	SEC/ (ms/cm)
R1	44.27	2.65	1.46	13.98	199.85	0.79	19.01	17.73	6.85	0.56
R2	32.45	0.85	0.61	18.20	44.85	17.45	54.29	11.78	8.28	0.56
R3	9.59	1.98	0.68	17.66	136.58	3.97	152.80	12.27	7.97	0.60
R4	22.78	1.32	0.52	19.34	89.09	0.93	21.39	18.19	8.08	0.61
R5	3.86	5.19	0.57	20.63	373.02	3.31	33.42	22.01	7.75	0.53
R6	30.93	3.09	0.51	19.51	213.33	3.16	66.06	12.29	7.48	0.62
R7	31.18	1.91	0.62	18.26	124.93	2.09	28.36	19.76	8.01	0.48
R8	3.65	0.48	0.37	18.37	21.18	0.51	18.44	7.81	8.36	0.50
R9	3.60	0.61	0.55	20.68	18.01	0.64	36.40	7.62	8.72	0.48
R10	43.62	2.54	0.92	19.44	158.57	1.18	135.01	12.83	7.84	0.60
R11	44.50	2.63	0.72	19.22	158.82	2.83	89.63	10.70	7.92	0.60
R12	16.13	1.12	0.61	18.58	70.91	0.37	84.83	6.95	7.95	0.60

2 Supplementary Figures

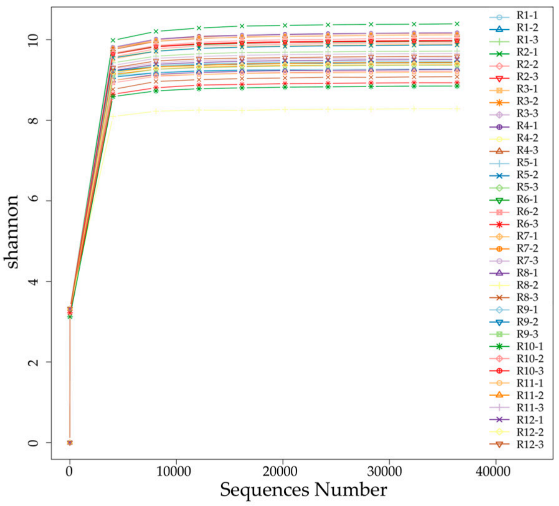

(a)

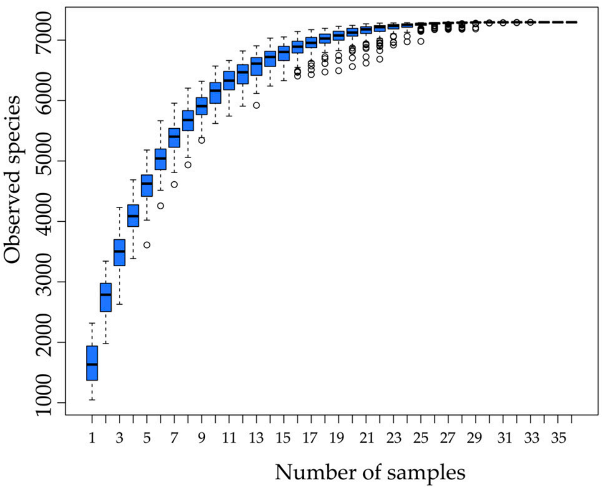

(b)

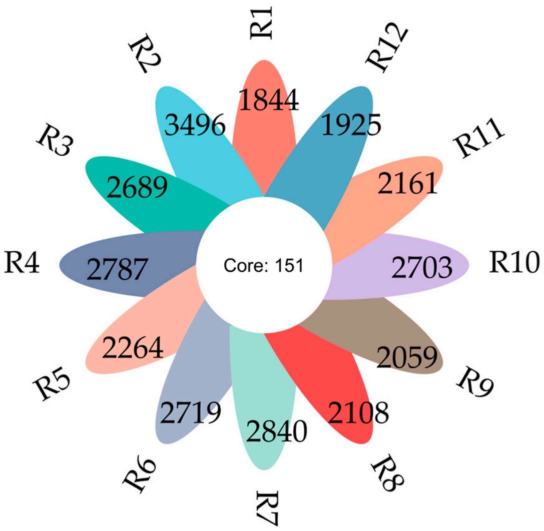

(c)

Supplementary Figure S1. Analysis of sequencing results quality and ASVs quantity of 12 habitats. **Note:** (a) Dilution curves, (b) Species cumulative box plots, (c) Venn diagrams.

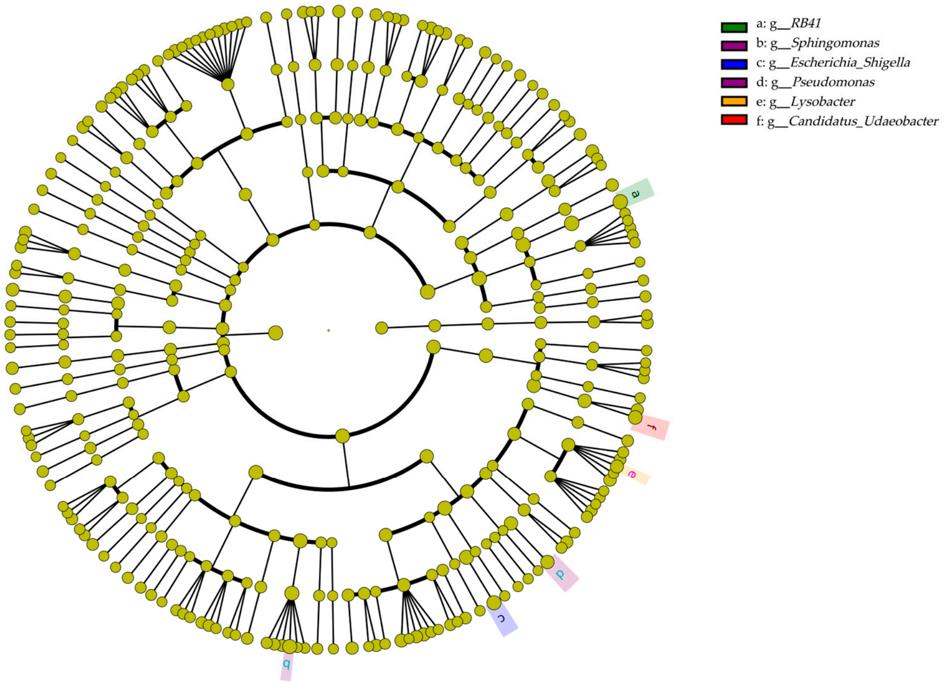

(a)

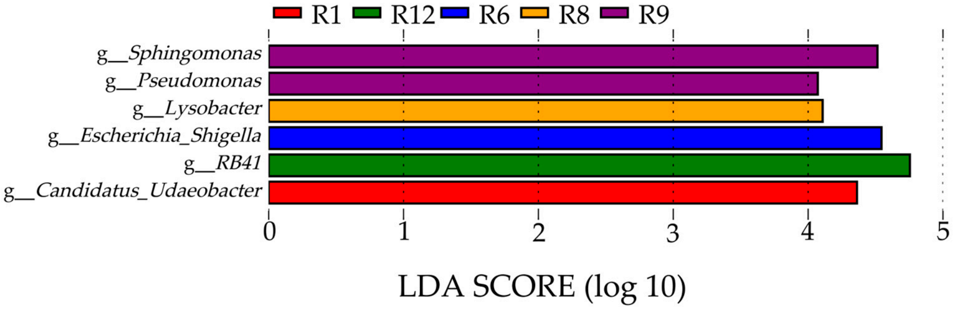

(b)

Supplementary Figure S2. LEfSe analysis of 12 habitats at genus level. **Note:** (a) LEfSe analysis (LDA > 4.0) , (b) LDA value of indicator species (LDA > 4.0).

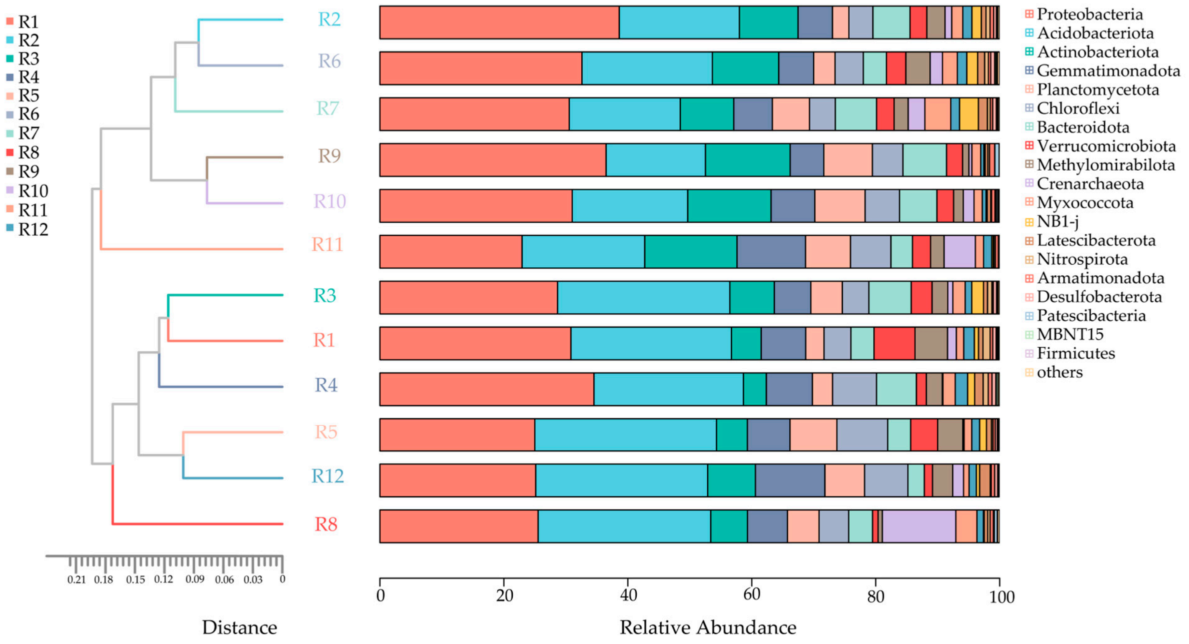

Supplementary Figure S3. UPGMA clustering tree based on weighted Bray-Curtis distance. **Note:** Left panel illustrates the UPGMA clustering tree structure; right panel presents the phylum-level relative abundance profile of soil bacteria.
